# Editorial: Neuronal cytoskeleton and GTPases in health and diseases

**DOI:** 10.3389/fcell.2022.1025527

**Published:** 2022-09-15

**Authors:** Cecilia Conde, Emily Anne Bates, Corina Garcia, Oscar M. Lazo

**Affiliations:** ^1^ Department of Neurobiology, Instituto de Investigación Médica Mercedes y Martín Ferreyra INIMEC-CONICET-UNC, Córdoba, Argentina; ^2^ University of Colorado Anschutz Medical Campus, Aurora, CO, United States; ^3^ IIBBA-CONICET Leloir Institute Foundation, Buenos Aires, Argentina; ^4^ UCL Queen Square Institute of Neurology and UK Dementia Research Institute, University College London, London, United Kingdom

**Keywords:** cytoskeleton, neuronal polarity, microtubules, neuronal development, synaptic plasticity (LTP/LTD), GTPases

## Introduction

The specialized cytoarchitecture of neurons enable the formation of complex neuronal networks, which play a fundamental role integrating the diversity of physiological systems in animals. The development and maintenance of these networks across the central and peripheral nervous system are essential to process sensory inputs and coordinate motor activity, as well as giving rise to memory, learning, and behavior. One of the crucial events during neuronal development is the break of cellular symmetry that leads to neuronal polarization (Dotti et al., 1988; Caceres et al., 2012). From that moment neurons develop a single axon and multiple dendrites, which creates two morphologically and functionally distinct domains providing compartmentalization and directionality to neuronal communication.

This complex neuronal morphology is largely driven by the cytoskeleton (Witte and Bradke, 2008), both as a structural determinant of the axonal and dendritic complexity, as well as a key regulator of directional transport of membrane organelles and molecular complexes along the neurites (Conde and Caceres, 2009; Koppers and Farias, 2021). These two complementary roles are regulated at different levels by constitutive properties of cytoskeletal proteins; actin- and microtubule-binding proteins modulating their dynamics, geometry and association with motors; as well as signaling molecules integrating intrinsic genetic programs and extrinsic cues in time and space, giving each neuron its specific morphology.

Ras superfamily of small GTPases represents one of the most diverse and influencing nodes of cytoskeletal regulation (Goitre et al., 2014; Qu et al., 2019; Song et al., 2019). Among its members, the Rho GTPases are major regulators of cytoskeleton dynamics, with essential functions in cell migration, proliferation, differentiation, polarity, vesicle and membrane trafficking, survival and apoptosis (Stankiewicz and Linseman, 2014; Aspenstrom, 2018; Guo et al., 2020). On the other hand, Rap GTPases have crucial signaling and cell adhesion roles in neurons (Shah and Puschel, 2016). Additionally, Arf and Rab GTPases are master regulators of vesicular and membrane trafficking (Kiral et al., 2018; Arrazola Sastre et al., 2021), mediating the recruitment of specific molecular motors, adaptors and cytoskeleton binding proteins to an enormous diversity of cargoes, including synaptic vesicles, signaling endosomes and mitochondria in axons and dendrites (Villarroel-Campos et al., 2014; Villarroel-Campos et al., 2018). Disrupted function of GTPases is associated with cancer, neurodevelopmental conditions and neurodegenerative diseases, making them particularly interesting as potential therapeutic targets (Aspenstrom, 2018; Costain et al., 2019; Qu et al., 2019; Guo et al., 2020).

In this Research Topic, we have collected new articles exploring different layers of this complexity, from Tubulin isoforms to GTPase effectors and activity during development, physiology, and disease. The original findings and novel integrative perspectives included in this Research Topic nicely illustrate the diversity of models and mechanisms where the most recent research on cytoskeleton and GTPases come together.

## Research

The group of Park et al., presents an original research article on tubulinopathies. A collaboration between clinicians and research scientists at the University of Colorado yielded important insights into how GTPase dependent conformational changes in tubulin stabilize microtubules for correct brain development. Mutations that result in a T178M substitutions disrupt TUBB2A and TUBB3 beta tubulin isotypes were identified in two different patients with severe brain malformations. In addition, a previous report had shown reduced myelination in the brain resulting from a T178M substitution in TUBB4A (Tonduti et al., 2016). Now Park et al., made the same substitution in yeast beta tubulin to determine the molecular consequences of T178M on tubulin, showing that T178M dominantly inhibits microtubule assembly and catastrophe by mimicking the GTP bound state of beta tubulin. The differences between T178M TUBB2A and T178M TUBB3 patient phenotypes support a model in which the timing and cell type of isotype expression dictates the tissue level consequences of a mutation in tubulin.

In a review that also links cellular and molecular mechanisms orchestrated by GTPases to a variety of human diseases, also including Paget’s disease of the bone and Chronic obstructive pulmonary disease, the work by Shen et al. provides a detailed and interesting panorama of the recently emerging evidence linking RIN3 (Ras and Rab interactor 3) to Alzheimer’s disease (AD) and other dementias. RIN3 has been previously identified as a risk factor for late onset AD (LOAD) based on a polymorphism within the enhancer region of the Rin3 gene (Lambert et al., 2013). The evidence reviewed by the authors suggests that gene variants of this guanine nucleotide exchange factor (GEF) would increase Rab5 activity, disrupting formation and axonal transport of endosomal compartments, therefore, contributing to early cellular pathogenesis in AD.

On a different note, Akagawa et al. contributed the Research Topic with an insightful piece proposing a novel cytoplasmic role for nuclear proteins, such as some cyclins, cyclin-dependent kinases (CDKs) and RAD21, as well as DNA repair proteins including BRCA2, p53 and RAD17, which have been found to be expressed in post-mitotic neurons. Consistent with these ideas, Cdk5 (an atypical neuronal CDK) is required for neuronal migration, dendrite formation, and synaptic plasticity through regulation of the neuronal cytoskeleton and membrane trafficking (Kawauchi, 2014). Surprisingly, some cell cycle related conventional CDKs (cyclin D2, Cdk4, 6, and 7) were also found to be expressed after neurogenesis. Moreover, expression of DNA repair proteins such as BRCA1/BRCA2/RAD51 has been observed in mouse cerebral cortices at embryonic day 10 and 18, as well as in 2 months old mice; whereas ablation of BRCA1 gene results in a severe disruption of the cortical layers. Several studies indicate that these nuclear proteins also indirectly interact with the cytoskeleton (Mejat and Misteli, 2010; Lottersberger et al., 2015). Thus, this extra-cell cycle regulatory or DNA repair function would expand the repertoire of cytoplasmic proteins identified as involved in the regulation of neuronal microtubules and the actin cytoskeleton, with crucial roles on migration, neuronal morphology, and synapses during different stages of nervous system development.

Finally, the Research Topic includes a revision of Rho GTPase signaling, presented by Zhang et al., focusing on Rho contribution to the mechanisms of synaptic plasticity and memory. The authors discuss recent works showing the participation of this family of GTPases in long-term potentiation (LTP) and long-term depression (LTD), as well as a key regulators of spine morphology. The work adds an interesting emphasis on the different roles that the members of Rho family play, Cdc42 being required for LTP induction, RhoA being more important for LTP maintenance, and Rac1 participating in the destabilization of LTP and induction of LTD. Accordingly, a tight association of these GTPases regulating molecular and cellular mechanisms of learning and memory is proposed: while Cdc42 and RhoA seem to be involved the formation of memory, Rac1 is more important in memory elimination. At the end of this article, the authors discuss the role of these GTPases in Alzheimer’s disease, Autism spectrum disorder and Fragile X syndrome, offering an interesting association between alterations of Rho function and brain disorders of learning and memory.

In summary, this Research Topic of articles for the Research Topic *Neuronal cytoskeleton and GTPases in health and diseases* provides good examples of the different layers of regulation of cytoskeleton dynamics by GTPases, as well as a valuable update on several aspects of their involvement in the initiation, maintenance, and regulation of different neuronal functions and pathological conditions.

## References

[B1] Arrazola Sastre A. Luque Montoro M. Lacerda H. M. Llavero F. Zugaza J. L. (2021). Small GTPases of the Rab and Arf families: Key regulators of intracellular trafficking in neurodegeneration. Int. J. Mol. Sci. 22, 4425. 10.3390/ijms22094425 33922618 PMC8122874

[B2] Aspenstrom P. (2018). Activated Rho GTPases in cancer-the beginning of a new paradigm. Int. J. Mol. Sci. 19, E3949. 10.3390/ijms19123949 30544828 PMC6321241

[B3] Caceres A. Ye B. Dotti C. G. (2012). Neuronal polarity: demarcation, growth and commitment. Curr. Opin. Cell Biol. 24, 547–553. 10.1016/j.ceb.2012.05.011 22726583 PMC3425660

[B4] Conde C. Caceres A. (2009). Microtubule assembly, organization and dynamics in axons and dendrites. Nat. Rev. Neurosci. 10, 319–332. 10.1038/nrn2631 19377501

[B5] Costain G. Callewaert B. Gabriel H. Tan T. Y. Walker S. Christodoulou J. (2019). De novo missense variants in RAC3 cause a novel neurodevelopmental syndrome. Genet. Med. 21, 1021–1026. 10.1038/s41436-018-0323-y 30293988

[B6] Dotti C. G. Sullivan C. A. Banker G. A. (1988). The establishment of polarity by hippocampal neurons in culture. J. Neurosci. 8, 1454–1468. 10.1523/jneurosci.08-04-01454.1988 3282038 PMC6569279

[B7] Goitre L. Trapani E. Trabalzini L. Retta S. F. (2014). The ras superfamily of small GTPases: the unlocked secrets. Methods Mol. Biol. 1120, 1–18. 10.1007/978-1-62703-791-4_1 24470015

[B8] Guo D. Yang X. Shi L. (2020). Rho GTPase regulators and effectors in autism spectrum disorders: Animal models and insights for therapeutics. Cells 9, E835. 10.3390/cells9040835 32244264 PMC7226772

[B9] Kawauchi T. (2014). Cdk5 regulates multiple cellular events in neural development, function and disease. Dev. Growth Differ. 56, 335–348. 10.1111/dgd.12138 24844647

[B10] Kiral F. R. Kohrs F. E. Jin E. J. Hiesinger P. R. (2018). Rab GTPases and membrane trafficking in neurodegeneration. Curr. Biol. 28, R471–R486. 10.1016/j.cub.2018.02.010 29689231 PMC5965285

[B11] Koppers M. Farias G. G. (2021). Organelle distribution in neurons: Logistics behind polarized transport. Curr. Opin. Cell Biol. 71, 46–54. 10.1016/j.ceb.2021.02.004 33706233

[B12] Lambert J. C. Ibrahim-Verbaas C. A. Harold D. Naj A. C. Sims R. Bellenguez C. (2013). Meta-analysis of 74,046 individuals identifies 11 new susceptibility loci for Alzheimer's disease. Nat. Genet. 45, 1452–1458. 10.1038/ng.2802 24162737 PMC3896259

[B13] Lottersberger F. Karssemeijer R. A. Dimitrova N. de Lange T. (2015). 53BP1 and the LINC complex promote microtubule-dependent DSB mobility and DNA repair. Cell 163, 880–893. 10.1016/j.cell.2015.09.057 26544937 PMC4636737

[B14] Mejat A. Misteli T. (2010). LINC complexes in health and disease. Nucleus 1, 40–52. 10.4161/nucl.1.1.10530 21327104 PMC3035119

[B15] Qu L. Pan C. He S. M. Lang B. Gao G. D. Wang X. L. (2019). The ras superfamily of small GTPases in non-neoplastic cerebral diseases. Front. Mol. Neurosci. 12, 121. 10.3389/fnmol.2019.00121 31213978 PMC6555388

[B16] Shah B. Puschel A. W. (2016). Regulation of Rap GTPases in mammalian neurons. Biol. Chem. 397, 1055–1069. 10.1515/hsz-2016-0165 27186679

[B17] Song S. Cong W. Zhou S. Shi Y. Dai W. Zhang H. (2019). Small GTPases: Structure, biological function and its interaction with nanoparticles. Asian J. Pharm. Sci. 14, 30–39. 10.1016/j.ajps.2018.06.004 32104436 PMC7032109

[B18] Stankiewicz T. R. Linseman D. A. (2014). Rho family GTPases: key players in neuronal development, neuronal survival, and neurodegeneration. Front. Cell. Neurosci. 8, 314. 10.3389/fncel.2014.00314 25339865 PMC4187614

[B19] Tonduti D. Aiello C. Renaldo F. Dorboz I. Saaman S. Rodriguez D. (2016). TUBB4A-related hypomyelinating leukodystrophy: New insights from a series of 12 patients. Eur. J. Paediatr. Neurol. 20, 323–330. 10.1016/j.ejpn.2015.11.006 26643067

[B20] Villarroel-Campos D. Gastaldi L. Conde C. Caceres A. Gonzalez-Billault C. (2014). Rab-mediated trafficking role in neurite formation. J. Neurochem. 129, 240–248. 10.1111/jnc.12676 24517494

[B21] Villarroel-Campos D. Schiavo G. Lazo O. M. (2018). The many disguises of the signalling endosome. FEBS Lett. 592, 3615–3632. 10.1002/1873-3468.13235 30176054 PMC6282995

[B22] Witte H. Bradke F. (2008). The role of the cytoskeleton during neuronal polarization. Curr. Opin. Neurobiol. 18, 479–487. 10.1016/j.conb.2008.09.019 18929658

